# Antidepressant-like activity of Bezafibrate in mice models of depression: a behavioral and neurobiological characterization

**DOI:** 10.3389/fphar.2025.1595341

**Published:** 2025-04-30

**Authors:** Dawei Xu, Jin Zhou, Siyi Zhou, Weizhen Wang, Chengniu Wang, Bo Jiang, Wei Zhao

**Affiliations:** ^1^ Department of Orthopaedics, Second Affiliated Hospital of Nantong University, Nantong, Jiangsu, China; ^2^ Institute of Reproductive Medicine, Medical College, Nantong University, Nantong, Jiangsu, China; ^3^ Department of Pharmacology, Pharmacy College, Nantong University, Nantong, Jiangsu, China; ^4^ Department of Neurosurgery, Second Affiliated Hospital of Nantong University, Nantong, Jiangsu, China

**Keywords:** Bezafibrate, depression, PPARα, BNDF, CUMS

## Abstract

**Background:**

Depression represents a major global public health challenge, inflicting profound suffering on patients while imposing substantial socioeconomic burdens on families and healthcare systems. Although monoamine-based antidepressants remain first-line pharmacotherapy, accumulating clinical evidence reveals several limitations of these medications, including delayed pharmacodynamics and low remission rates. Therefore, it is necessary to search for new drugs and develop effective strategies for depression treatment. Bezafibrate (BEZ), which can activate proliferator-activated receptor a (PPARα), exhibit various biological functions, such as improving mitochondrial function, reducing neuroinflammation, and improving cognitive function. This study is to explore whether BEZ has antidepressant-like effects and its potential mechanisms.

**Methods:**

The antidepressant effects and potential mechanisms of BEZ were assessed by using forced swim test, tail suspension test, sucrose preference test, Western blot, gene interference, and immunofluorescence in the chronic unpredictable mild stress (CUMS) models of depression.

**Results:**

Results showed that BEZ treatment significantly reversed depressive behavior in CUMS mice. The administration of BEZ obviously promoted the expression of PPAR, enhanced the BDNF signaling pathway, promoted hippocampal neurogenesis in CUMS mice. In addition, the pharmacologcial inhibitors GW6471 and K252a were obviously prevented the antidepressant effect of BEZ. Furthermore, gene knockdown of hippocampal PPARα or BDNF by using AAV-PPARα-shRNA-EGFP and AAV-BDNF-shRNA-EGFP, can remarkably inhibit the antidepressant effect of BEZ.

**Conclusion:**

Collectively, the behavioral and neurobiological results demonstrate that BEZ exhibits antidepressant-like activity through PPARα/BDNF signaling pathway and may use as a potential antidepressant.

## 1 Introduction

Currently, depression has become a prevalent psychiatric disorder, affecting over 300 million individuals worldwide and significantly compromising quality of life and functional capacity (Meller et al., 2021; Pano et al., 2021; Miola et al., 2023). This debilitating condition, characterized by notably high recurrence rates (60%–80%) and elevated suicide risk (Xu et al., 2017; Monroe and Harkness et al., 2022), manifests core symptoms including persistent low mood and social withdrawal (Wu et al., 2022; Zhang et al., 2024c). Beyond emotional disturbances, depression profoundly impacts cognitive processes, behavioral patterns, and physical health status (Khazanov et al., 2022; Prizeman et al., 2023). Although monoamine-based antidepressants remain the primary pharmacological intervention in clinical practice, their therapeutic utility is substantially limited by delayed onset of action, suboptimal response rates, and unsatisfactory remission rates (Blier and Mansari, 2013; Dale et al., 2015). Therefore, searching for new therapeutic targets and developing new antidepressant drugs are very meaningful.

Research shows that neurotrophic factors are very important for the central nervous system (Popova et al., 2017). Brain derived neurotrophic factor (BDNF), an important neurotrophic factor expressed in the cerebral cortex and hippocampus, has been proven to be an antidepressant target (Duman, 2004; Björkholm and Monteggia, 2016; Zhang et al., 2016a). Studies have shown that BDNF and its downstream molecules transmembrane protein receptor tyrosine kinase B (TrkB) and cAMP-response element binding protein (CREB) play a key role in the pathophysiology of depression (Wang et al., 2022b; Ye et al., 2024). BDNF binds to its receptor TrkB, activating the BDNF/TrkB signaling pathway and further leading to phosphorylation of the transcription factor CREB (Du et al., 2020). Research has found that the phosphorylated CREB in the hippocampus is downregulated in patients with severe depression (Guan et al., 2021). The downregulation of BDNF-CREB signaling in the hippocampus is considered a major factor in triggering depression (Wu et al., 2022). Therefore, developing new antidepressant drugs targeting BDNF in the hippocampus as a therapeutic target is a highly effective strategy.

Previous studies have shown that hippocampal peroxisome proliferator-activated receptor a (PPARα) is a potential target of new antidepressants (Zhang et al., 2023b; Alzarea and Rahman, 2025). Some studies researches have shown that venlafaxine and vortioxetine exert antidepressant effects through hippocampal PPARα (Chen et al., 2019; Wang et al., 2017c). Reports suggest that the PPARα pathway may be involved in the therapeutic potential of N-palmitoylethanolamide for depressive mood disorders (Zhang et al., 2023b). Bezafibrate (BEZ), which can activate PPARα with ability to decrease triglyceride and increase high density lipoprotein- cholesterol, is used as a lipid-lowering agent in clinical practice (Tenenbaum and Fisman, 2012). It can reduce triglyceride, cholesterol levels, blood viscosity, and improve endothelial function (Ohno et al., 2014). Recent studies have shown that BEZ has neuroprotective effects, such as reducing neuroinflammation and improving cognitive/memory function (Dumont et al., 2012; Lu et al., 2023). In addition, studies have shown that BEZ has a certain preventive effect on emotional disorders (Wang et al., 2017c). Based on this, we speculate that BEZ has antidepressant function. To prove our hypothesis, we investigated the possible antidepressant effects of BEZ using the chronic unpredictable mild stress (CUMS) models of depression in this study. The results demonstrate that BEZ exhibits antidepressant-like activity through PPARα/BDNF signaling pathway. This study would extend the understanding of BEZ’s pharmacological activities and may provide a novel antidepressant candidate.

## 2 Materials and methods

### 2.1 Animals

Male C57BL/6 J mice (8 weeks old) were sourced from the Experimental Animal Center of Nantong University. All animals were randomized into different experimental groups based on their weight. Mice were maintained under controlled environmental conditions: 12:12 h light-dark cycle (07:00–19:00 illumination phase), ambient temperature regulated at 22°C–24°C, and relative humidity stabilized at 55% ± 10%. Standard rodent chow and autoclaved water were provided *ad libitum* throughout the acclimatization and experimental periods. All protocols were conducted in strict compliance with the Institutional Animal Care and Use Committee guidelines of Nantong University, under ethical approval certificate no. S20240709-002 (Jiangsu Province Animal Care Ethics Committee).

### 2.2 Materials

BEZ and fluoxetine hydrochloride were sourced from Sigma-Aldrich (St. Louis, MO, United States). Both compounds were dissolved in 0.5% carboxymethylcellulose sodium (CMC-Na) vehicle solution, with control groups receiving equivalent volumes of CMC-Na alone. Dose selection for BEZ (25, 50, 100 mg/kg, i.p.) was based on established previous reports (Dumont et al., 2012), while the fluoxetine dosage (20 mg/kg, i.p.) followed previously validated antidepressant protocols (Xu et al., 2017). During the final 14 days of chronic unpredictable mild stress (CUMS) exposure, mice received daily intraperitoneal injections of either vehicle, fluoxetine, or BEZ at designated concentrations prior to behavioral assessments. GW6471 was purchased from Tocris (Bristol, UK). K252a was purchased from Sigma-Aldrich (St. Louis, MO, United States). The primary antibodies for PPARα, BDNF, pAKT, pTrkB, total AKT, total TrkB, and β-actin, were obtained from Abcam (Cambridge, UK).

### 2.3 Chronic unpredictable mild stress

The mice were exposed to a variable sequence of unpredictable mild stressors (CUMS) for 8 weeks, which was performed as described in previous work (Xu et al., 2017; Wu et al., 2022). A total of eight different stressors were randomly adopted including shaking (30 min), restraint (1 h), 4°C exposure (1 h), cage tilting (12 h), day/night inversion, water deprivation (23 h), food deprivation (23 h) and damp bedding (24 h). Administration of BEZ/fluoxetine/vehicle was performed daily in the final 2 weeks during the whole CUMS period. Then forced swim test, tail suspension test and sucrose preference test with a randomized double-blind were perform together to assay the CUMS-induced depressive-like behaviors in mice.

### 2.4 Forced swim test

Forced swim test (FST) were conducted as previously described (Wu et al., 2022), which was used to evaluate despair-like behavior in mice. In briefly, each mouse was individually placed in a transparent cylinder (25 cm height, 20 cm diameter) containing water (25°C ± 1°C, 15 cm height) and forced to swim for a 6 min. The water in the cylinders was changed after each test. The immobility duration of each mouse was recorded and hand-scored for the last 4 min and considered to be the status when the mouse was floating in the water with the absence of struggle, or making only those movements necessary to keep breath.

### 2.5 Tail suspension test

The tail suspension test (TST) was conducted to evaluate antidepressant-like activity following established methodologies (Wang et al., 2020a). Mice were securely affixed to the suspension a tabletop using adhesive tape applied 1 cm from the distal tail tip. Subjects were individually suspended 60 cm above the testing surface for a 6-minute observation period. Immobility time, defined as the duration of passive hanging without voluntary body movement, was recorded and subsequent behavioral analysis.

### 2.6 Sucrose preference test

Sucrose preference test (SPT) was used for evaluating anhedonia in mice. It was performed as previous described (Wu et al., 2022; Wang et al., 2020a). Firstly, the mice received sucrose preference training for 2 days before the experiment. Then, after being deprived of water for 18 h, each mouse was given with two pre-weighed bottles complemented with water or 1% sucrose solution (w/v) for 6 h. The liquid consumption of each mouse was weighed and recorded. The sucrose preference ratio (SP%) was calculated as (sucrose water consumption (g)/(sucrose consumption + water consumption (g)) × 100%.

### 2.7 Western blot analysis

The Western blotting procedures have been frequently described in our previous reports (Xu et al., 2017). The mice were anesthetized with carbon dioxide, then euthanized by cervical dislocation, and the brain was directly separated and collected using surgical forceps using anatomical methods. With carbon dioxideThe hippocampi tissues of each mouse were rapidly dissected and lysed in ice with NP-40 lysis buffer (150 mM NaCl, 1% IGEPAL, 50 mM Trizma base (Sigma-T4661, pH 8.0) containing 1 mM phenyl-methyl-sulfonyl fluoride (PMSF). After centrifugation, the protein supernatant was collected and determined the protein concentrations by BCA method. The protein supernatant was mixed with 4×loading buffer and deactivated in 95°C for 5 min. After that, SDS-PAGE (sodium dodecyl sulfate-polyacrylamide gel electrophoresis) was used to separate proteins with different molecular components, and then transferred to a polyvinylidene difluoride (PVDF) membrane. Primary antibodies used to recognize specific protein, including: PPARα (1:500), BDNF (1:500), TrkB (1:1000), phospho-TrkB-Tyr515 (pTrkB; 1:500), ERK (1:1000), phospho-ERK-Thr202/Tyr204 (pERK1/2; 1:500), AKT (1:1000), phospho-AKT-Ser473 (pAKT; 1:500), CREB (1:1000), phospho-CREB-Ser133 (pCREB; 1:500) and β-actin (1:2000) were used. After washing with Tris Buffered Saline with Tween three times, the membranes were incubated with IR-Dye 680-labeled secondary antibodies (1:5000; Licor, Lincoln, United States) for 1 h at room temperature. An Odyssey CLx detection system was adopted for scanning.

### 2.8 Hippocampal injection of PPARα-shRNA-EGFP and BDNF-shRNA-EGFPRNA sequencing and data analysis

The production of AAV-PPARα-shRNA-EGFP, AAV-BDNF-shRNA-EGFP, and AAV-Control-shRNA-EGFP has been described in a previous reports (Wu et al., 2022; Wang et al., 2021d). Briefly, each animal was anesthetized with 0.5% pentobarbital sodium and fixed in a stereotactic frame. After cutting open the scalp, drill a small hole in the skull of each mouse, a 10 μL Hamilton syringe was positioned at the hippocampus coordinates: AP = −2.3 mm, ML = ± 1.6 mm, DV = + 1.8 mm. AAV-PPARα-shRNA, AAV-BDNF-shRNA, or AAV-Control-shRNA (5 × 10^12^ TU/mL) was bilaterally infused into the hippocampus region of each mouse using the syringe at a rate of 0.5 μL/min (1.5 μL/each side). After the infusion, the syringe was left in place for 5 min before being retracted slowly. The wound of each mouse was cleaned and sutured. Two weeks was required for the expression of AAV to be stable in the hippocampus. The nucleotide sequences for PPARα-shRNA, BDNF-shRNA and Control-shRNA were 5′-AGA​AAT​TCT​TAC​CTG​TGA​A-3′, 5′-TGAGCGTGTGTGA CAGTATTA-3′ and 5′-TTCTCCGAACGTGTC ACGT-3′, respectively (Wu et al., 2022; Wang et al., 2021d).

### 2.9 Immunofluorescence

As we have frequently described (Wu et al., 2022). After anaesthesia with carbon dioxide, the mice were transcardially perfused with normal saline (4% NaCl) followed by 4% paraformaldehyde (PFA) in 0.1 M phosphate buffer (PBS), and then hippocampal slices were postfixed for 24 h in 4% PFA at 4°C. Next, the hippocampal slices were dehydrated in 30% sucrose solution (48 h, 4°C) until sinking to the bottom of the 50 mL tube and cut (25 µm) using a freezing microtome (Leica, Wetzlar, Germany). Sections of selected areas were blocked by incubation in PBS plus 0.3% Triton X-100 for 30 min at room temperature (RT) and subsequently incubated with 3% bovine serum albumin for 30 min at RT. Then, the slices were incubated in primary antibody against doublecortin (DCX, 1:100; Cell signaling) overnight at 4°C and then washed in PBS. Incubated slices were then incubated in FITC-labeled secondary antibody (1:50; Pierce, Rockford, IL, United States) for 2 h at RT and then washed in PBS. Last, the slices were incubated with DAPI for 10 min at RT and washed again. The sections were mounted on slides and coverslipped. All images were obtained using FLUOVIEW FV1200 confocal microscopes (Olympus) and Olympus VS200. Digitalized images were analyzed using Fuji (NIMH, Bethesda MD, United States). The quantification method has also been described in our previous report (Lu et al., 2023). Examination of the DCX-positive (DCX^+^) cells were confined to the dentate gyrus (DG), in particular the granule cell layer (GCL), including the subgranular zone (SGZ) of hippocampus that defined as a two-cell body-wide zone along the border between the GCL and the hilus. Quantifications of the DCX^+^ cells were respectively conducted from 1-in-6 series of hippocampal sections spaced at 150 μm and spanning the rostrocaudal region of the DG bilaterally. Every DCX^+^ cell within the GCL and SGZ was counted.

### 2.10 Statistical analysis

Statistical analyses were performed using GraphPad Prism 7.0 (GraphPad Software, Inc., La Jolla, CA, United States). The differences between mean values were evaluated using One-way ANOVA (*post hoc* Tukey’s test). Data are expressed as the mean ± S.E.M. P < 0.05 is considered as statistical significant.

## 3 Results

### 3.1 BEZ treatment has antidepressant like effects in the CUMS model of depression in FST, TST and SPT

**FIGURE 1:**
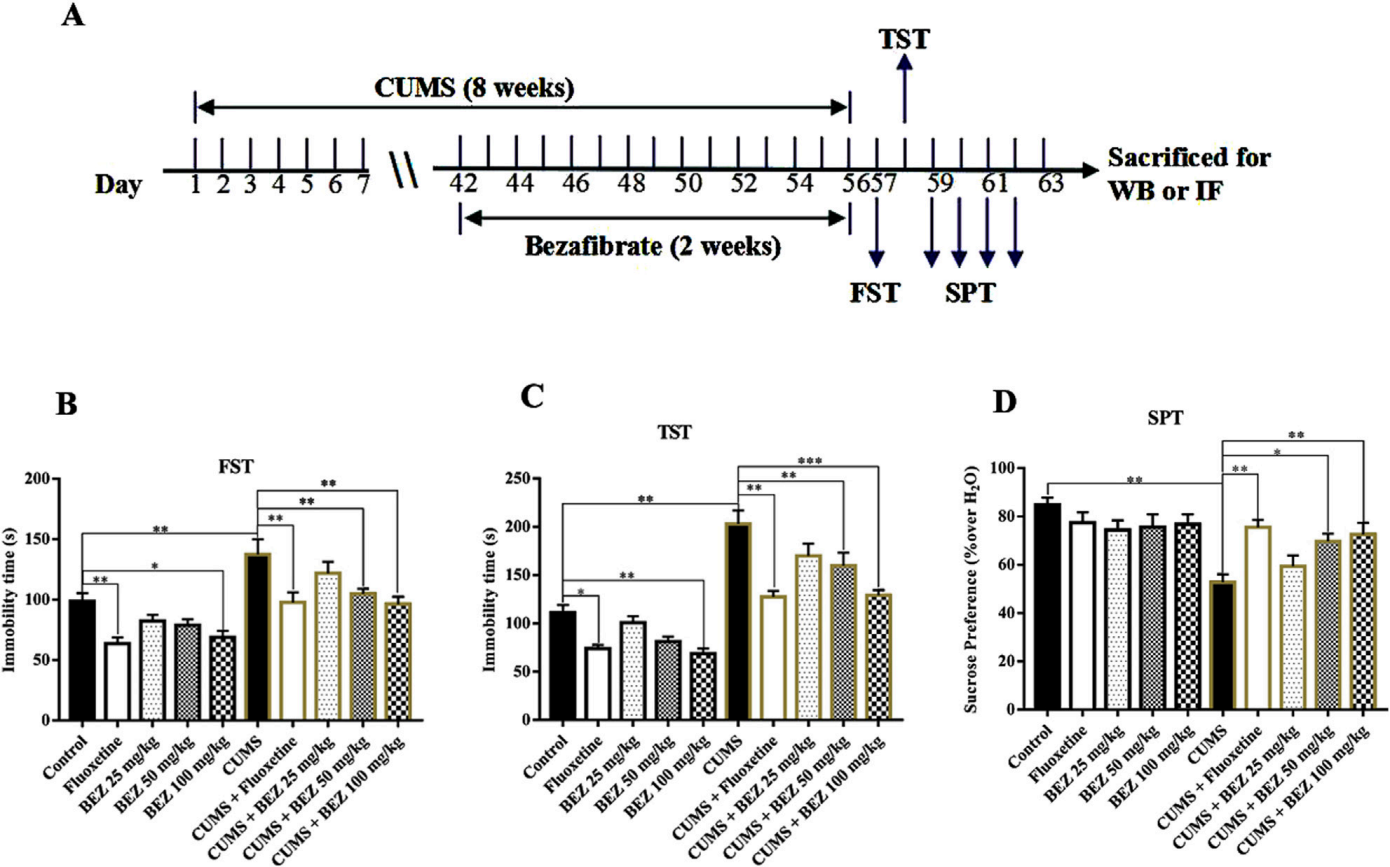
BEZ treatment significantly improves depressive behaviors in CUMS-induced mice. **(A)** Schematic timeline of the experimental procedures. **(B)** The immobility duration of FST was obviously decreased in the CUMS-induced mice after BEZ treatment. **(C)** The immobility duration of TST was notably reduced in the CUMS-induced mice after BEZ treatment. **(D)** The sucrose preference was significantly increased in the CUMS-induced mice after BEZ treatment. *P < 0.05, **P < 0.01, ***P < 0.001, n = 10 biological replicates.

In order to evaluate the potential antidepressant effect of BEZ, we conducted TST, FST, and SPT experiments in the CUSM of model depression. Fluoxetine, a classical antidepressant which enhances serotoninergic neurotransmission through potent and selective inhibition of neuronal reuptake of serotonin (Benfield et al., 1986), was used as a positive control in our study. As shown in Figure 1B, the FST results show that the mice immobility was significantly increased in the CUMS compared the control group (P = 0.003). The results also reveal that administration of (20 mg/kg) fluoxetine obviously reduced the mice immobility time, and (25, 50, and 100 mg/kg) BEZ reduced the mice immobility time in a concentration dependent manner in the CUMS. The administration of 50 and 100 mg/kg BEZ notably shortened the mice immobility time compared with the CUMS group (P = 0.02, 0.001, respectively, Figure 1B). The TST experiment showed similar results, compared with the control group, the immobility time in the TST was obviously increased in the CUMS mice, and decreased by fluoxetine and BEZ treatment, respectively. The administration of 50 and 100 mg/kg BEZ notably shortened the mice immobility time in the TST compared with the CUMS group (P = 0.008, <0.001, respectively, Figure 1C). As shown in Figure 1D, the SPT displayed that CUMS obviously decreased the sucrose preference compared with the control group, and 50 and 100 mg/kg BEZ significantly improve the mice sucrose preference in the CUMS model of depression (P = 0.034, = 0.007, respectively, Figure 1D). These results indicate that BEZ has potential antidepressant like effects.

### 3.2 BEZ treatment promotes the expression levels of the hippocampal PPARα and BDNF signaling pathway in the CUMS mice

To investigate the underlying antidepressant mechanism of action of BEZ, the BDNF signaling pathway in the hippocampus of mice was tested by Western blotting. The high concentrations of BEZ (100 mg/kg) was selected for further research. As shown in Figure 3, the expression of hippocampal PPARα in the CUMS group was significant decreased compared with the control group (P < 0.001). And the protein level of PPARα/β-actin was obviously increased after BEZ treatment compared with the CUMS group (P < 0.001). The results of Western blotting also revealed significant decease protein levels of BDNF/β-actin (P < 0.001), pTrkB/TrkB (P < 0.001), pAKT/AKT (P < 0.001), pERK/ERK(P < 0.001), and pCREB/CREB (P < 0.001) in the CUMS group compared that in the control group. And the protein levels of BDNF/β-actin (P < 0.001), pTrkB/TrkB (P < 0.001), pAKT/AKT (P = 0.006), pERK/ERK (P < 0.001), and pCREB/CREB (P < 0.001) were significantly increased in the CUMS mice after BEZ treatment. Meanwhile, our study found no significant changes in the total levels of TrkB, AKT, ERK, and CREB proteins in hippocampus. These results suggest that BEZ may exert antidepressant effects through the BDNF signaling pathway.

### 3.3 BEZ treatment promotes hippocampal neurogenesis in the CUMS mice

Depression not only leads to dysfunction of the BDNF system, but also accompanies a decrease in hippocampal neurogenesis, which can be reversed through antidepressant treatment. We further studied the effect of BEZ on hippocampal neurogenesis through immunofluorescence staining. As shown in Figure 3, the DCX fluorescence staining results showed a significant decrease in the number of DCX positive cells in the DG region of CUMS model mice compared to the control group (p < 0.001). After BEZ administration, the number of DCX positive cells significantly increased (p < 0.001). Meanwhile, BEZ administration significantly promoted the number of DCX positive cells. Collectively, BEZ treatment significantly improved hippocampal neurogenesis in CUMS mice.

### 3.4 Blocking hippocampal PPARα or BDNF signaling pathway fully blocks the antidepressant effect of BEZ in the CUMS mice

BEZ may produce antidepressant like effects by activating the PPARα and BDNF system based on above results. To further confirm this hypothesis, GW6471, a pharmacological inhibitor of PPARα, and K252a, a pharmacological inhibitor of TrkB, were used (Dumont et al., 2012; Pereira and Hiroaki-Sato, 2018). The CUMS-treated mice were co-injected with BEZ (100 mg/kg) + GW6471 (1 mg/kg), or BEZ (100 mg/kg) + K252a (25 μg/kg) during the last 2 weeks. As shown in Figure 4, GW6471 or K252a alone did not affect the immobility time in the FST and TST in the CUMS mice, while they significantly inhibited the antidepressant effect of BEZ in FST (CUMS + BEZ + GW6471 vs. CUMS + BEZ, P = 0.012, CUMS + BEZ + K252a vs. CUMS + BEZ, P = 0.025) and TST (CUMS + BEZ + GW6471 vs. CUMS + BEZ, P = 0.031, CUMS + BEZ + K252a vs. CUMS + BEZ, P = 0.032), respectively. In addition, both GW6471 and K252a obviously prevented the antidepressant effect of BEZ on the sucrose preference by SPT (CUMS + BEZ + GW6471 vs. CUMS + BEZ, P = 0.002, CUMS + BEZ + K252a vs. CUMS + BEZ, P = 0.001) in the CUMS mice.

Furthermore, we selectively knockdown the hippocampal expression of PPARα and BDNF by using AAV-PPARα-shRNA-EGFP and AAV-BDNF-shRNA-EGFP, respectively. Briefly, mice brain-stereotactic injection with PPARα-shRNA or BDNF-shRNA, then subjected to CUMS and BEZ (100 mg/kg) treatment. As shown in Figure 5, stable expression of AAV in the hippocampus, which confirmed the silencing efficacy of PPARα-shRNA and BDNF-shRNA (Figures 5A,B). The behavioral tests were performed by FST, TST and SPT (Figures 6, 7). The results indicated that PPARα-shRNA can remarkably inhibit the decreasing effect of BEZ on FST immobility time (CUMS + BEZ + PPARα-shRNA vs. CUMS + BEZ, P = 0.009) and TST immobility time (CUMS + BEZ + PPARα-shRNA vs. CUMS + BEZ, P = 0.02), and PPARα-shRNA also obviously prevented the enhancing effects of BEZ on the sucrose preference of CUMS mice (CUMS + BEZ + PPARα-shRNA vs. CUMS + BEZ, P = 0.023) (Figure 6). Similarly, the results showed that the usage of BDNF-shRNA evidently blocked the decreasing effects of BEZ treatment on the FST immobility time (CUMS + BEZ + BDNF-shRNA vs. CUMS + BEZ, P = 0.05) and TST immobility time (CUMS + BEZ + BDNF-shRNA vs. CUMS + BEZ, P = 0.037), and BDNF-shRNA also significantly prevented the enhancing effects of BEZ on the sucrose preference of CUMS mice (CUMS + BEZ + BDNF-shRNA vs. CUMS + BEZ, P = 0.011) (Figure 7).

## 4 Discussion

At present, depression seriously limits the psychosocial function of patients and reduces the quality of life, while bringing huge economic burden to families and society (Malhi and Mann, 2018; Pereira and Hiroaki-Sato, 2018). Antidepressants are one of the main treatment methods for depression, and in clinical practice, they often produce therapeutic responses by increasing the synaptic concentration of monoamine neurotransmitters, but the response and remission rates of these drugs are relatively low (Wu et al., 2022). Therefore, it is necessary to explore new antidepressant targets and drugs at present. Currently, exploring potential new uses of known drug provides a new solution for the high investment and low output dilemma encountered in new drug research and development. BEZ is a lipid-lowering drug used in clinical practice (Monk and Todd, 1987). Recently, studies have shown that BEZ also has neuroprotective effects and a certain preventive effect on emotional disorders (Dumont et al., 2012; Lu et al., 2023; Wang et al., 2017c). We speculate that BEZ may have antidepressant-like function. To our knowledge, this is the first study on the antidepressant effect of BEZ, and we found that BEZ treatment induced notable antidepressant efficacy in the CUMS depression model. In addition, the promotion of hippocampal BDNF signaling cascade and neurogenesis is associated with the antidepressant like efficacy of BEZ, and both the pharmacological blockade and genetics blockade of the BDNF system significantly inhibits the antidepressant effect of BEZ. Overall, our findings expand our knowledge of the pharmacological effects of BEZ and provide a new potential antidepressant.

The FST and TST are two commonly used detection methods for screening antidepressant drugs. In these two tests, rodents are in an inescapable stress environment and will become helpless and immobile after the initial period of struggle, which is similar to human depression and can be reversed by antidepressants (Yankelevitch-Yahav et al., 2015). CUMS is an animal model widely used in depression research, which can induce behavioral and neurobiological changes in rodents, resemble clinical depression in humans (Antoniuk et al., 2019). The results display that BEZ significantly shortened the immobility time of mice in both FST and TST, increased the sucrose preference in a concentration dependent manner (25, 50, 100 mg/kg) in CUMS mice (Figure 1). And the antidepressant effect of 100 mg/kg BEZ is similar to that of fluoxetine, a clinical antidepressant. These results suggest that BEZ may provide new drugs for the treatment of depression in the future.

**FIGURE 2:**
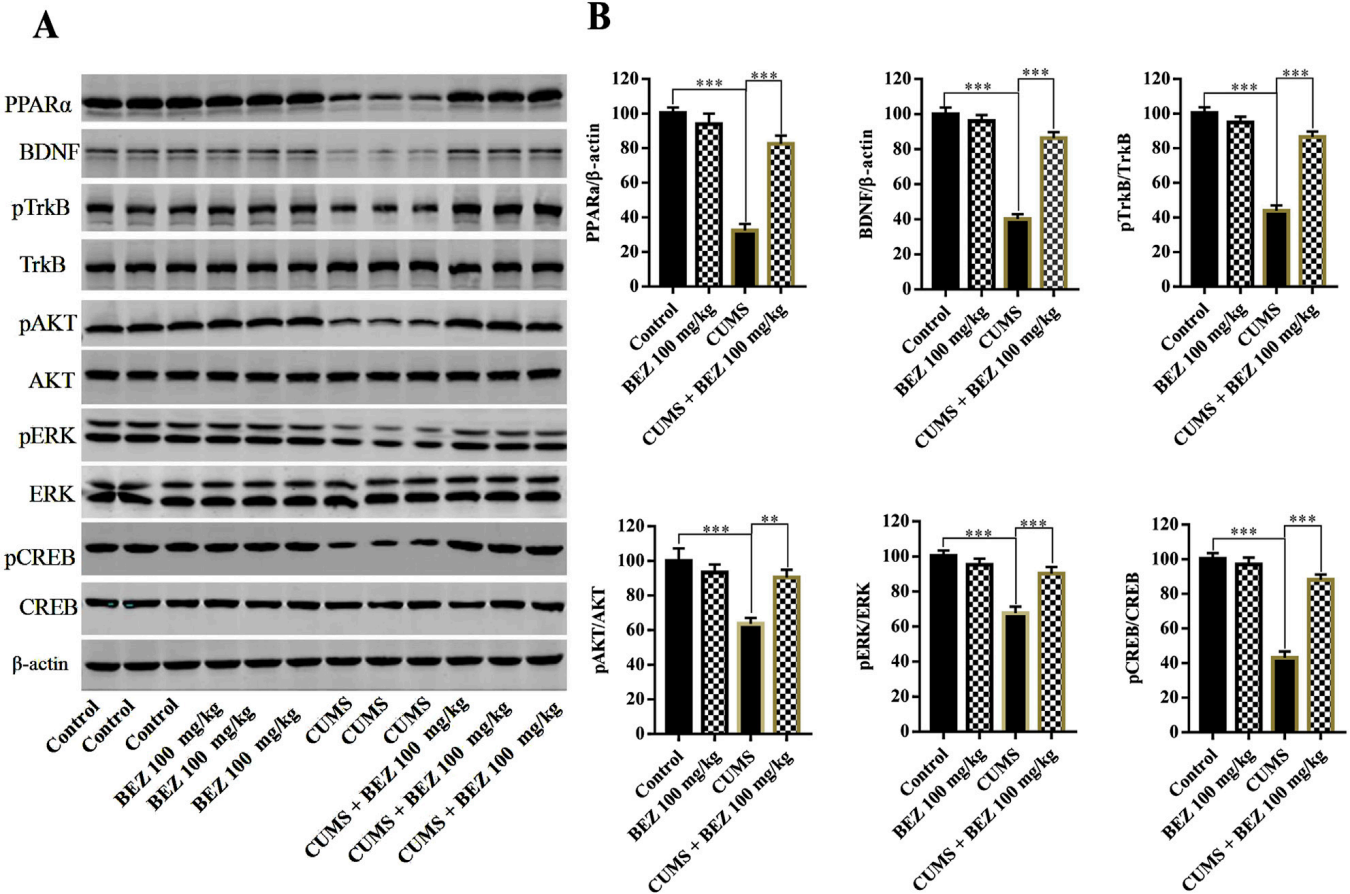
BEZ administration promotes the expression levels of the hippocampal PPARα and BDNF signaling pathway in CUMS-induced mice. **(A)** The expression levels of PPARα, BDNF, pTrkB, TrkB, pAKT, AKT, pERK, ERK, pCREB, and CREB were quantified by Western blot analysis. **(B)** The statistical analysis of protein levels. **P < 0.01, ***P < 0.001, n = 5 biological replicates.

**FIGURE 3:**
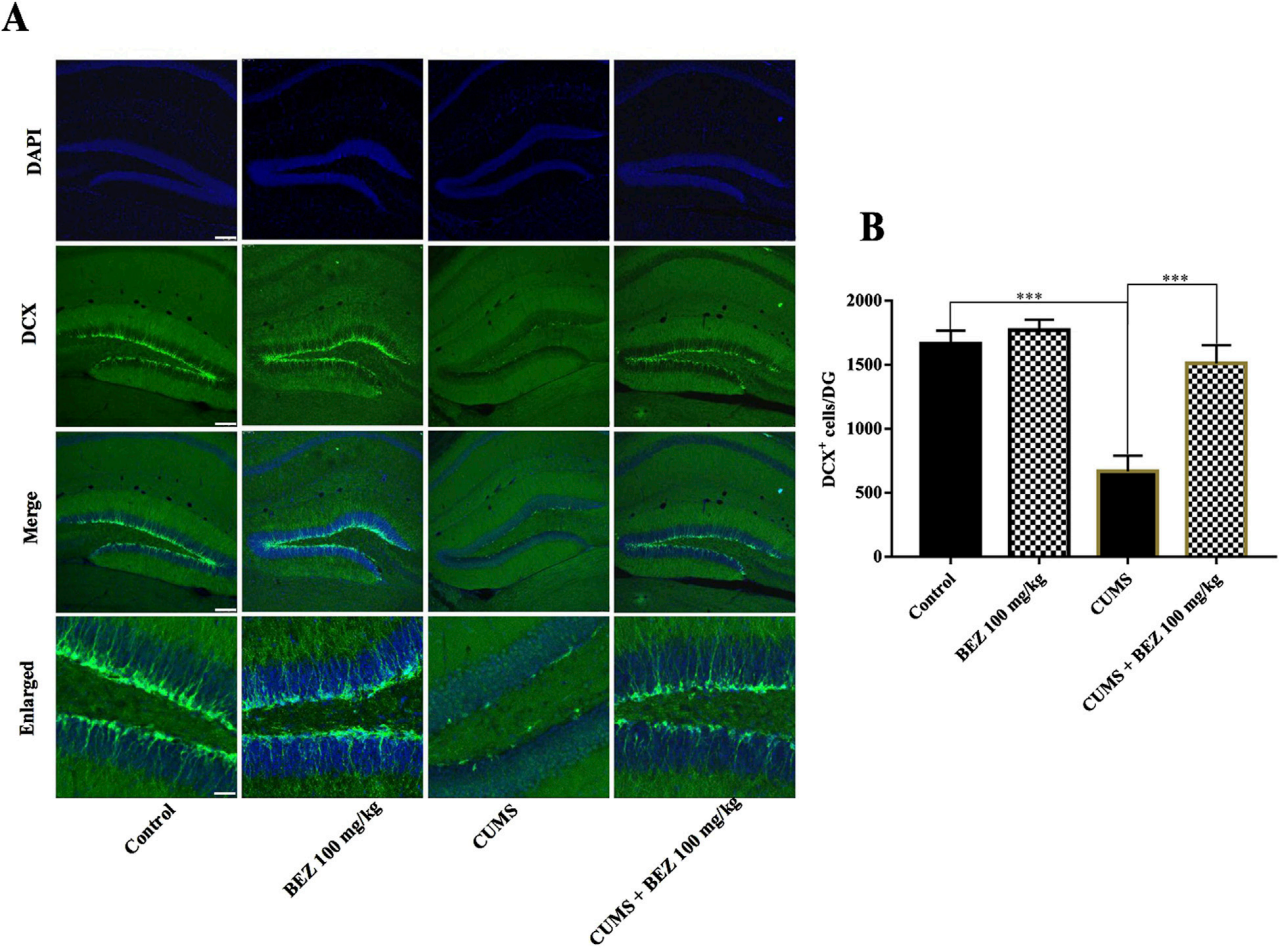
BEZ treatment promotes hippocampal neurogenesis in the CUMS-induced mice. **(A)** Immunofluorescent staining of DCX in the DG region, Scale bar = 150 μm. **(B)** Representative images of confocal microscopy and corresponding analyses of the number of DCX positive cells in the DG region. ***P < 0.001, n = 5 biological replicates.

**FIGURE 4:**
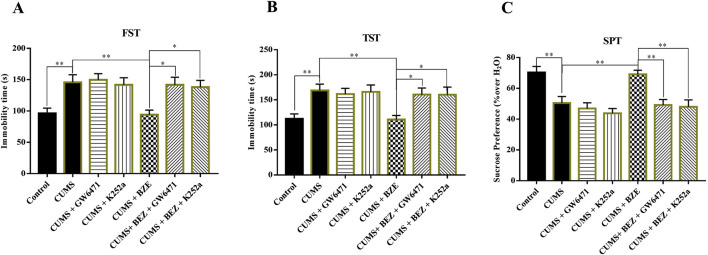
Blockade of the PPARα and BDNF signaling pathway by GW6471 and K252a abolished the antidepressant efficacy of BEZ in mice. **(A)** Mice in the (CUMS + BEZ + GW6471)-treated and (CUMS + BEZ + K252a)-treated group spent significantly more time being immobile than mice in the (CUMS + BEZ)-treated groups in the FST. **(B)** Mice in the (CUMS + BEZ + GW6471)-treated and (CUMS + BEZ + K252a)-treated group spent significantly more time being immobile than mice in the (CUMS + BEZ)-treated groups in the TST. **(C)** Mice in the (CUMS + BEZ + GW6471)-treated group and (CUMS + BEZ + K252a)-treated group displayed notably lower sucrose preference than mice in the (CUMS + BEZ)-treated group. *P < 0.05, **P < 0.01, n = 10 biological replicates.

**FIGURE 5:**
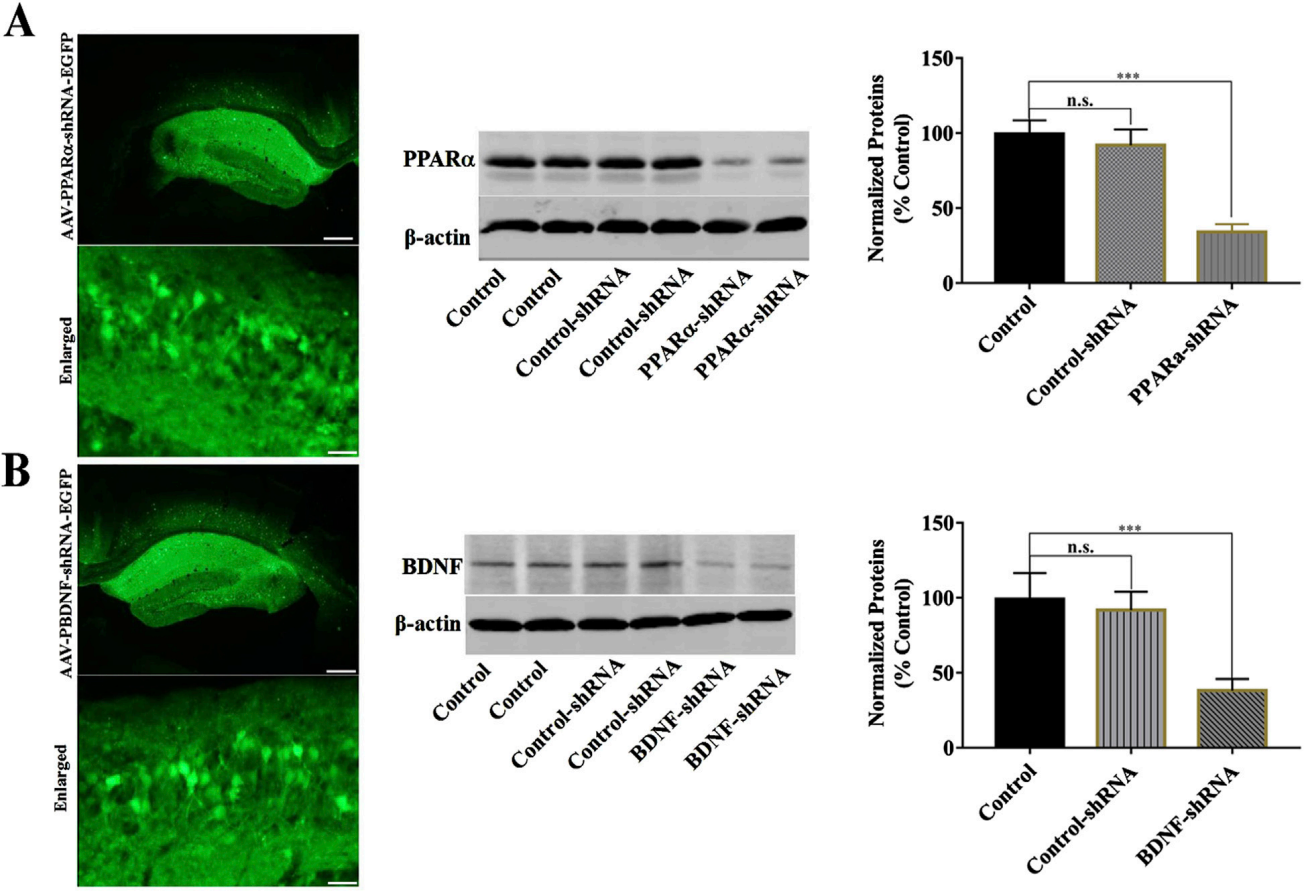
Hippocampal PPARα-knockdown by PPARα-shRNA and BDNF-knockdown by BDNF-shRNA. **(A)** Fluorescence images of a fixed hippocampal slice which expressed AAV-PPARα-shRNA-EGFP 2 weeks after its stereotactic infusion. The scale bars of representative and enlarged images are 400 and 50 μm, respectively. The following Western blotting results confirmed the silencing effects of PPARα-shRNA on the protein expression of hippocampal PPARα. ***P < 0.001, n = 5 biological replicates. **(B)** Fluorescence images of a fixed hippocampal slice which expressed AAV-BDNF-shRNA-EGFP 2 weeks after its stereotactic infusion. The scale bars of representative and enlarged images are 400 and 50 μm, respectively. The following Western blotting results confirmed the silencing effects of BDNF-shRNA on the protein expression of hippocampal BDNF. ***P < 0.001, n = 5 biological replicates.

**FIGURE 6:**
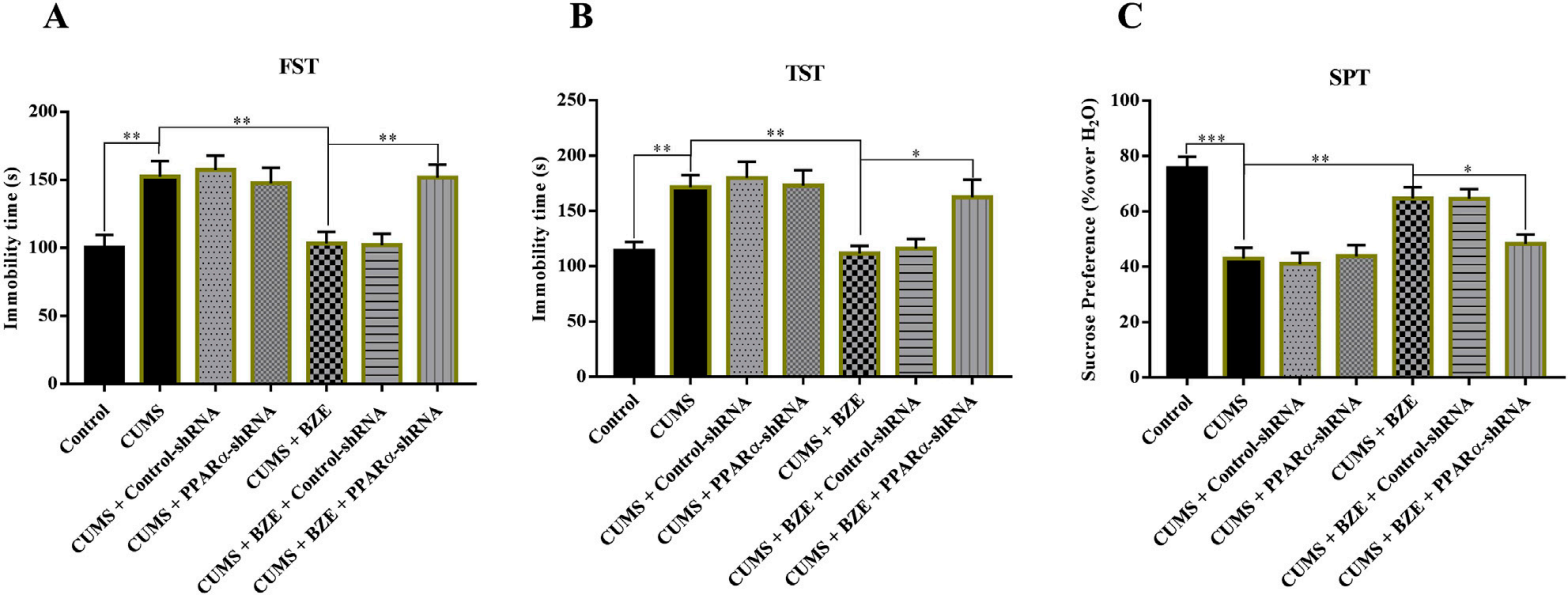
Hippocampal PPARα-knockdown by PPARα-shRNA abolished the antidepressant activity of BEZ in mice. **(A)** Mice in the (CUMS + BEZ + BDNF-shRNA)-treated group spent significantly more time being immobile than mice in the (CUMS + BEZ)-treated and (CUMS + BEZ + control-shRNA)-treated groups in the FST. **(B)** Mice in the (CUMS + BEZ + PPARα-shRNA)-treated group spent significantly more time being immobile than mice in the (CUMS + BEZ)-treated and (CUMS + BEZ + control-shRNA)-treated groups in the TST. **(C)** Mice in the (CUMS + BEZ + PPARα-shRNA)-treated displayed notably lower sucrose preference than mice in the (CUMS + BEZ)-treated and (CUMS + BEZ + control-shRNA)-treated groups. *P < 0.05, **P < 0.01, n = 10 biological replicates.

**FIGURE 7:**
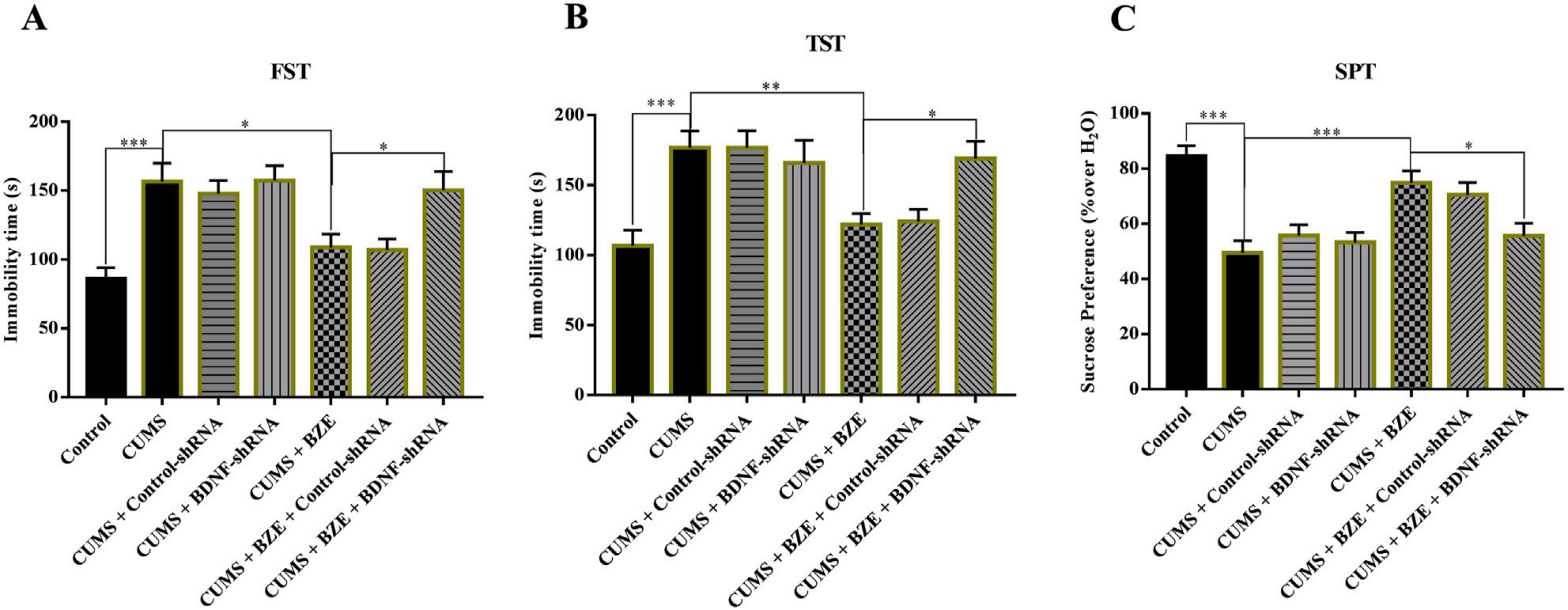
Hippocampal BDNF-knockdown by BDNF-shRNA abolished the antidepressant activity of BEZ in mice. **(A)** Mice in the (CUMS + BEZ + BDNF-shRNA)-treated group spent significantly more time being immobile than mice in the (CUMS + BEZ)-treated and (CUMS + BEZ + control-shRNA)-treated groups in the FST. **(B)** Mice in the (CUMS + BEZ + BDNF-shRNA)-treated group spent significantly more time being immobile than mice in the (CUMS + BEZ)-treated and (CUMS + BEZ + control-shRNA)-treated groups in the TST. **(C)** Mice in the (CUMS + BEZ + BDNF-shRNA)-treated displayed notably lower sucrose preference than mice in the (CUMS + BEZ)-treated and (CUMS + BEZ + control-shRNA)-treated groups. *P < 0.05, **P < 0.01, n = 10 biological replicates.

The BDNF protein in the hippocampus of the central nervous system is considered to be one of the important targets for antidepressant therapy (Vaidya and Duman, 2001; Malhi and Mann, 2018). Members of the BDNF signaling pathway such as TrkB, AKT, ERK, and CREB play critical roles in the pathophysiology of depression (Gong et al., 2024; Qin et al., 2024; Mohammadi et al., 2023; Matin and Dadkhah, 2024). Researches show that PPARs have multiple physiological functions, including regulating energy metabolism, anti-inflammatory, and neuroprotective functions (Scheggi et al., 2022; Titus et al., 2024). Currently, three PPAR isoforms have been identified, including PPAR-β/δ, and -γ. Reports show that PPARs have certain therapeutic prospects in psychiatric disorders (Rolland et al., 2013). Some research show that the overexpression of PPARγ in hippocampus protects mice against depression like behaviors induced by chronic stress (Liu et al., 2017). Studies suggest that PPARγ agonists may have antidepressant properties (Colle et al., 2017). The augmentation of PPARγ can have a positive impact on various important pathological processes of depression (Gold, 2021). Due to the structural similarity of PPAR isomers, PPARα may have antidepressant targets similar as PPAR-β/δ, and -γ. In addition, antidepressant effect was observed after administration with several PPARα agonists in some clinical studies (Jiang et al., 2015; Gao et al., 2022). In our study, the results show that BEZ significantly increase the expression of hippocampal PPARα, and enhance the expression of BDNF/TrkB/AKT/ERK/CREB signaling pathway in the CUMS mice (Figure 2). Furthermore, the immunofluorescence reveal that BEZ also significantly promoted neurogenesis in the hippocampus (Figure 3). These results suggest that BEZ may promote neurogenesis through PPARα/BDNF signaling pathway. In addition, pharmacological inhibition of GW6471 and K252a blocked the antidepressant effect of BEZ, which proved that BEZ was antidepressant through PPARα/BDNF signaling pathway (Figure 4). And the use of PPARα-shRNA and BDNF-shRNA effectively blocked the antidepressant effect of BEZ (Figures 5–7), which further confirms that the antidepressant effect of BEZ is mediated through the hippocampal PPARα/BDNF signaling pathway.

In our study, BEZ exerts antidepressant effects by activating PPARα/BDNF, but as pan PPAR agonists, it cannot be ruled out whether PPAR-β/δ, and -γ are involved in the antidepressant effects of BEZ. The limitations of this study include the fact that our research on the antidepressant effects of BEZ has not yet been investigated in other models of depression such as chronic restraint stress (CRS) models of depression and chronic social defeat stress (CSDS) models of depression. Moreover, the lipid-lowering effect of BEZ may independently affect stress response or neurogenesis. In addition, the CUMS protocol includes stressors such as food/water deprivation, which may interact with the metabolic processes of BEZ and potentially affect behavioral outcomes. This will be the focus of our next work. In addition, PPARα is implicated in many other CNS disorders, such as Alzheimer’s disease (Wójtowicz et al., 2020; Luo et al., 2020), Parkinson’s disease (Titus et al., 2024). BEZ, a PPARα agonist, has been used in clinical practice and may be used in the treatment of Alzheimer’s disease, Parkinson’s disease. Collectively, the results demonstrate that BEZ exhibits antidepressant-like activity through PPARα/BDNF signaling pathway in CUMS mice. Our study has proved for the first time that BEZ has the potential to be an antidepressant.

In this study, we demonstrated that a dose of 100 mg/kg of BEZ was optimal to improve oligozoospermia in mice. Utilizing the body surface area normalization method, we extrapolated a human equivalent dose of approximately 8 mg/kg, equivalent to a human daily oral dose of 560 mg for a 70 kg individual (Reagan-Shaw et al., 2008). Clinical studies reported that the long-term follow-up dose of BEZ in the treatment of myopathic carnitine palmitoyltransferase 2 deficiency was 600 mg daily (Bonnefont et al., 2010). This proves the safety of BEZ at the current dosage, further supporting the prospect of BEZ for the treatment of clinical depression.

## 5 Conclusion

Collectively, this study indicate that BEZ possesses antidepressant effects in mice which are mediated by activation of hippocampal PPARα/BDNF signaling pathway, thus providing the first evidence that BEZ can be a potential antidepressant candidate.

## Data Availability

The original contributions presented in the study are included in the article/supplementary material, further inquiries can be directed to the corresponding authors.
